# Spectral, thermal, antimicrobial studies for silver(I) complexes of pyrazolone derivatives

**DOI:** 10.1186/s13065-020-00723-0

**Published:** 2020-12-05

**Authors:** Soha F. Mohamed, Wesam S. Shehab, Aboubakr M. Abdullah, Mostafa H. Sliem, Walaa H. El-Shwiniy

**Affiliations:** 1 Department of Chemistry, Faculty of Science, Zagazig University, Zagazig, 44519 Egypt; 2 Center for Advanced Materials, Qatar University, P.O. Box 2713, Doha, Qatar; 3 Department of Chemistry, College of Science, University of Bisha, Bisha, 61922 Saudi Arabia

**Keywords:** Pyrazolones, Ag(i) complexes, Knoevenagel condensation, Antimicrobial activity

## Abstract

**Background:**

Synthesize new complexes of Ag(I) to enhance efficacy or stability and also, pharmacological activities on the operation of pyrazolone's biological properties.

**Results:**

Efficient and high yielding pathways starting from the versatile and readily available 3-methyl-1-phenyl-5-pyrazolone by Knoevenagel condensation of a sequence of 4-arylidene-3-methyl-1-phenyl-5-pyrazolone derivatives (**2a-c**) have been formed by the reaction of various substituted aromatic aldehydes Used as ligands to synthesize Ag(I) chelates. Synthesized compounds and their complexes have been characterized by elemental analysis, magnetic and spectroscopic methods (IR, ^13^C, ^1^HNMR, mass) and thermal analysis. The spectrophotometric determinations suggest distorted octaedral geometry for all complexes. Both ligands and their metal complexes have also been tested for their antibacterial and antifungal efficacy.

**Conclusions:**

Newly synthesized compounds have shown potent antimicrobial activity. The results showed that the complex 's high activity was higher than its free ligands, and that Ag(I)-L_3_ had the highest activity.

## Introduction

Pyrazolone chemistry began in 1883 when Ludwig Knorr first reacted to phenyl hydrazine with aceto-acetate ester. As pyrazolones were discovered as binding components for azo dyes in the late 1800s, they rapidly increased in importance. Today, pyrazolon is still an significant trade precursor to dyes and pharmaceuticals. Pyrazolone is a biologically important scaffold associated with different pharmacological activities such as antimicrobials [1–5], anti-inflammatory [6], analgesic [7], antidepressant [8], anticonvulsant [9], antidiabetic [10], antihyperlipidemic [11, 12], antiviral [13, 14], anti-tuberculosis [15, 16], antioxidant [17, 18] and anticancer [19, 20]. For several years, the preparation of pyrazolone and its derivatives has attracted significant attention from organic and medicinal chemists, as they belong to a class of compounds with promising results in medicinal chemistry. The heterocycles condensed to the pyrazole ring are an important source of bioactive molecules [21, 22]. Compounds containing both pyrazole and other essential heterocyclic active structural units usually demonstrate more remarkable biological activity. A number of condensed pyrazole derivatives have been reported as four-fold antibacterial agents against Gram-positive and Gram-negative bacteria compared to general pyrazole compounds [23, 24]. A digit of antimicrobial active silver(I) complexes have the capacity to disrupt microbial transpiration as well as block tyrosinase synthesis and are extremely cytotoxic to cancer cells [24]. Massive attention in silver ions (Ag(I)) as a broad spectrum antimicrobial has upped the size and importance of in vitro biocompatibility research [25]. Silver ions are toxic to many bacteria, viruses, algae and fungi. Silver-based medicines have been widely used for this task for decades [26]. The objective of this study is to display the synthesis and characterization of three Ag(I) pyrazolone complexes in an attempt to verify the mode of coordination and the biological properties of the final complexes.

## Results and discussion

### Synthesis and formulation

**Scheme 1:**
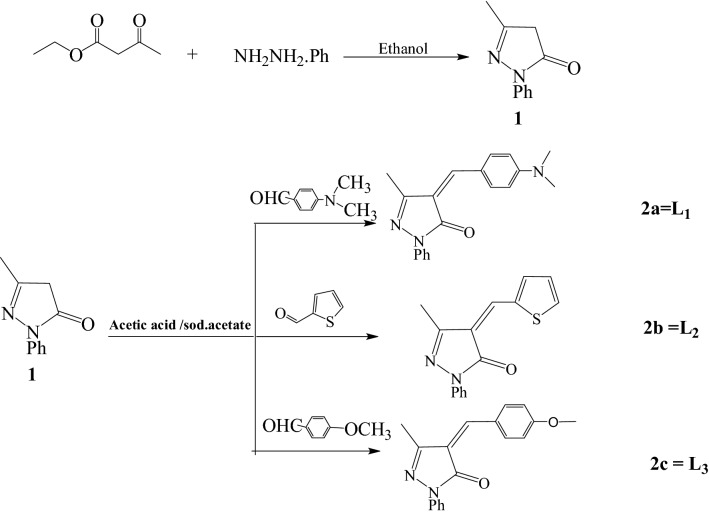
Synthesis of 4-arylidene-3-methyl-1-phenyl-5-pyrazolone derivatives

**Scheme 2:**
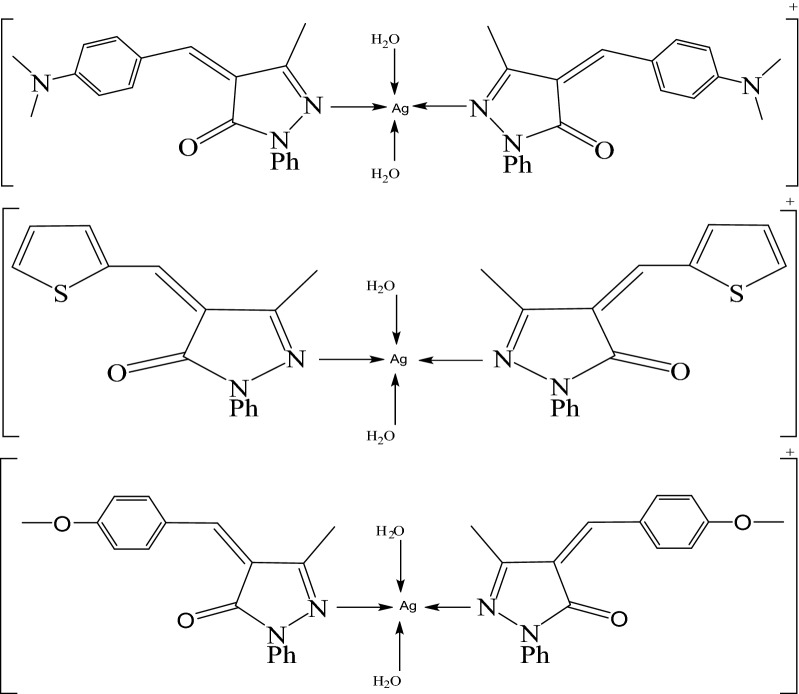
The coordinationn mode of Ag (I) with three ligand

A sequence of derivatives of 4-arylidene-3-methyl-1-phenyl-5-pyrazolone (4-(4-dimethylamino benzylidene)-3-methyl-1-phenyl-1*H*-pyrazol-5(4*H*)-one (2a) L_1_, 4-(4-Thiophene)-3-methyl-1-phenyl-1*H*pyrazol-5(4*H*)-one (2b) L_2_, 4-(4-methoxy benzylidene)-3-methyl-1-phenyl-1*H*pyrazol-5(4*H*)-one (2c) L_3_) is synthesized by condensing 3-methyl-1-phenyl-5-pyrazolone with substituted aromatic aldehydes as shown in Scheme 1 [27]. Three Ag(I) complexes have been prepared with the L_1_, L_2_, L_3_ ligands as shown in Scheme 2. Based on physicochemical and spectral data (IR and ^1^HNMR), structure of the synthesized compounds (**2a-c, Ag(I) complexes**) has been evaluated.

#### Infrared spectra

KBr disks registered mid-infrared spectra of L_1_, L_2_, L_3_ and their metal complexes. As expected, with changes in band intensities and wave numbers, the absorption bands characteristic of L_1_, L_2_, L_3_ acting as a monodentate unit are observed in the complexes. The proposed structures of the complexes must be considered prior to determining the assignments of the infrared spectra. Here, Ag(I) ion interacts with these monodentate ligands forming monomeric structure complexes in which the Ag(I) ion is four coordinated (Scheme 2) [27–30].

**Fig. 1:**
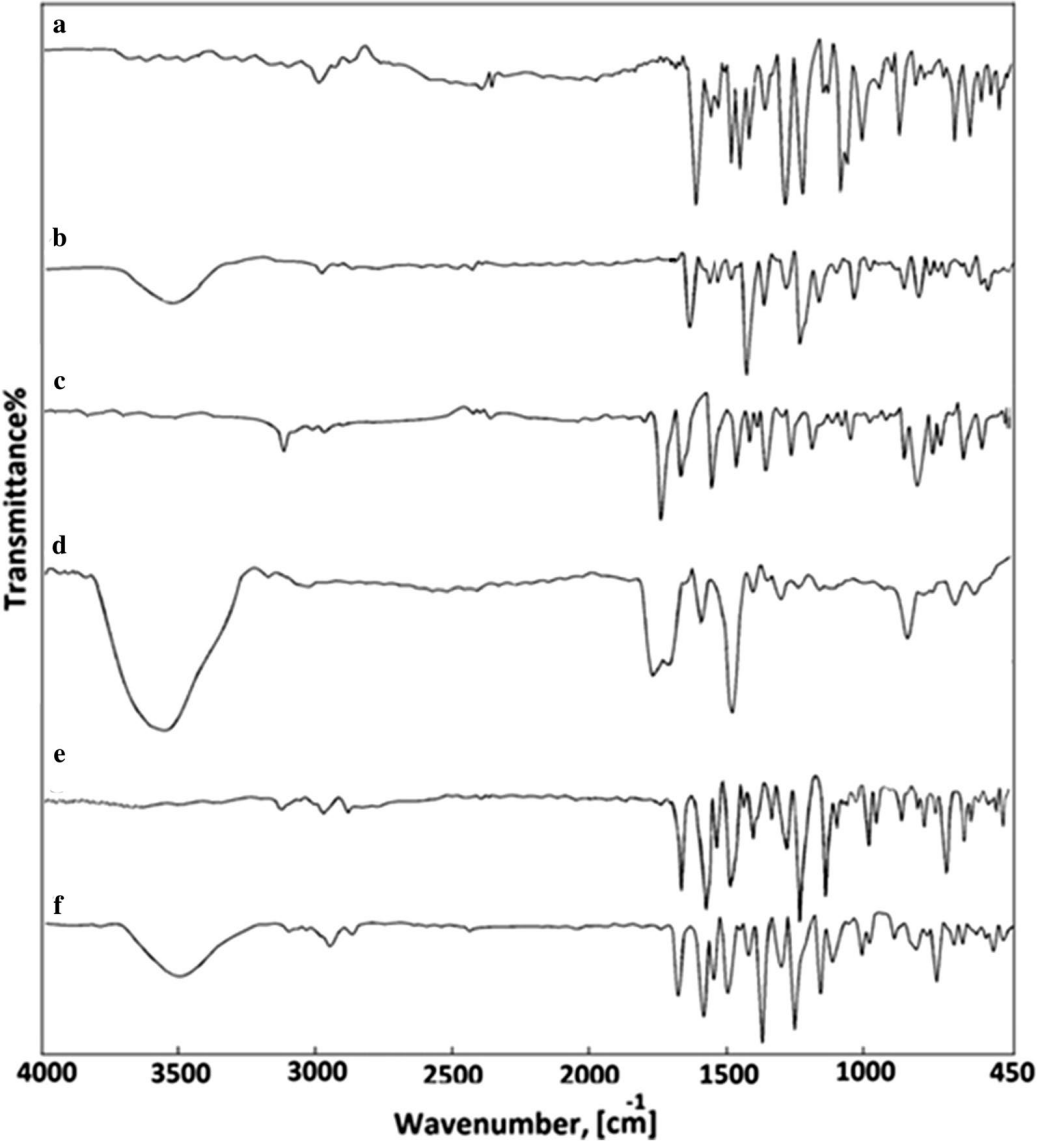
Infrared spectra for **a** L_1_, **b** [Ag(L_1_)_2_(H_2_O)_2_]NO_3_, **c** L_2_, **d** [Ag(L_2_)_2_(H_2_O)_2_]NO_3_.H_2_O, **e** L_3_ and **f** [Ag(L_3_)_2_(H_2_O)_2_]NO_3_

The complexes of three ligands with Ag(I) contain only one plane of symmetry and therefore the complexes that belong to C_S_ symmetry and show 159 vibrational fundamentals, and all vibrations are distributed between movements of the types A^\^ and A_1_^\\^, all of which are monodegenrate, infra—red and Raman active. The free ligand infrared spectrum shows bands at 1496, 1508 and 1550 cm^−1^ due to the stretching vibration of hydrazono (C = N) groups [31]. Comparing the Ag(I) IR spectrum with the free ligand spectrum, the transfer of (C = N) groups to lower frequency values (1512, 1515, 1523 and 1527 cm^−1^) and the change in strength of (C = N) from medium to strong (Fig. 1 and Table 1) which confirms that the ligand molecule coordinated with metal ions through the hydrazon nitrogen atom [31]. A medium wide band for the H_2_O stretching vibrations of coordinated water molecules at 3379, 3364, and 3364 cm^−1^ [31];The stretching vibrations ν(C-H) of phenyl groups and −CH_3_ units in these complexes are assigned as a number of bands in the region 3066–3100 cm^−1^ [11, 12]. The ν(C = O) vibration appears in the region of 1666–1685 cm^−1^. The spectra of the isolated solid complexes revealed a number of new bands of different intensities for ν(M–N). The ν(Ag–N) bands observed at 813, 837 cm^−1^ for Ag(I)-L_1_, at 748, 794 cm^−1^ for Ag(I)-L_2_ and at 759, 779 cm^−1^ for Ag(I)-L_3_ (Table 1) which are absent in the spectrum of free three ligands [30–32]. The coordinating water in the three complexes are characterized by the appearance of *ν*(Ag–O) at 577, 515, 544 cm^−1^. Also the stretching vibrations at 813, 792, 779 cm^−1^ assigned to *ν*(Ag OH_2_), sponsored coordinating water participation [32]. The suggested structural formulas are defined in Scheme 2 on the basis of the IR tests.

**Table 1:** Infrared frequencies (cm^−1^)^a^ and tentative assignments^b^ for (A) L_1_, (B) [Ag(L_1_)_2_(H_2_O)_2_]NO_3_, (C), L_2_ (D) [Ag(L_2_)_2_(H_2_O)_2_]NO_3_.H_2_O, (E) L_3_ and (F) [Ag(L_3_)_2_(H_2_O)_2_]NO_3_

A	B	C	D	E	F	Assignments
-	3379_m,br_	–	3364_m,br_	–	3364_m,br_	ν(O–H); coordinate H_2_O
3100_w_	3100_w_	3066_w_	3100_w_	3099	3100	ν(C-H); aromatic
2900	2900	2890	2885	2901	2900	ν(C-H); aliphatic
1670_ms_	1666_m_	1681_s_	1685_s,sh_	1678_m_	1678_m_	ν(C = O)
1550_s_ 1400_m_	1523_m_ 1410_s_	1496_m_ 1408_m_	1527_m_ 1381	1508_s_ 1427_vw_	1520_s_ 1415	ν(C = N) ν(C = C)
1319_s_ –	1319_s_ 1188_s_	1300_s_ -	1311_w_ 1165_m_	1311_sh_ -	1311_m_ 1172_s_	δ_b_(-CH_2_), ν(NO_3_^−1^)
1122_s_ 1018_w_ –	1122_s_ 1018_w_ –	1130_m_ – 1056_w_	1104_vw_ – 1099_sh_	1110_w_ – –	1130_m_ – –	ν(C–C), ν(C-N) ν(C = S)
954_w_ 943_vw_	995_w_ 995_m_	991_s_ 921_w_	941_vw_ 910_vw_	988_w_ 938_ s_	985_w_ 965_sh_	−CH-bend; phenyl
–	813_m_	–	792 m	–	779_m_	ν(Ag ←OH_2_)
–	577w	–	515w	–	544w	ν(Ag–O)
–	524w	–	498w	–	488w	ν(Ag–N)

^a^s = strong, w = weak, sh = shoulder, v = very, br = broad, ^b^ν = stretching and δ = bending

#### UV–Visible Spectra

**Fig. 2:**
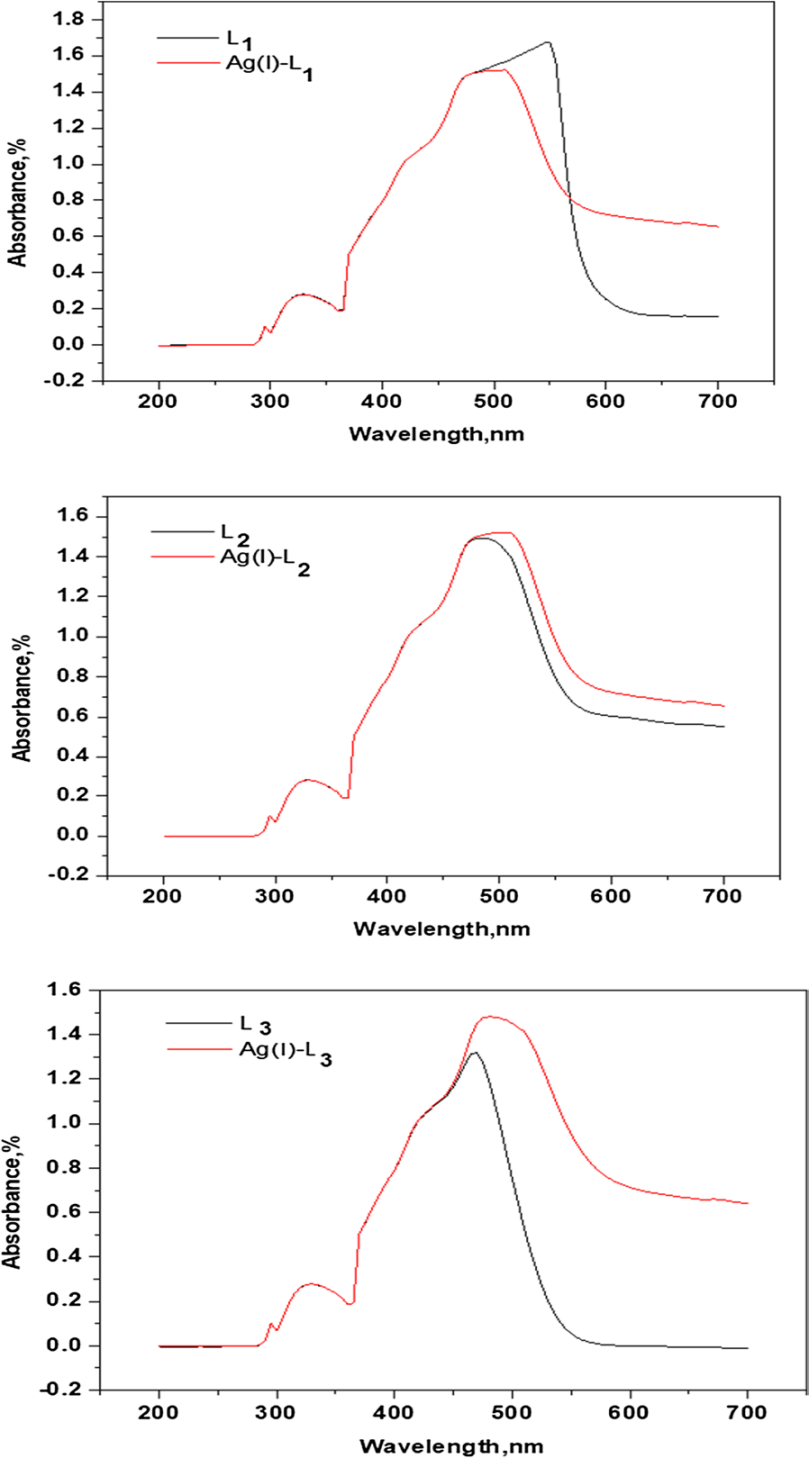
Electronic absorption spectra for L_1_, [Ag(L_1_)_2_(H_2_O)_2_]NO_3_, L_2_, [Ag(L_2_)_2_(H_2_O)_2_]NO_3_.H_2_O, L_3_ and [Ag(L_3_)_2_(H_2_O)_2_]NO_3_

The application of ultraviolet spectroscopy is more general and can be useful for all chelate structural determinations as they are all absorbed in this region [33]. Electronic absorption spectra confirmed the development of metal ligand complexes. Electronic absorption spectra L_1_ for Ag(I), L_2_ for Ag(I) and L_3_ for Ag(I). Complexes within the spectrum of wavelengths between 200 and 800 nm are described in Additional file 1: Table S1 and Fig. 2. The free three-ligand UV spectrum (L_1_, L_2_ and L_3_) displays bands at 281, 297 and 297 nm that are assigned respectively to π-π^*^. And displays bands allocated to n-π * transitions at 330 nm. The modification of the reflectance band to higher (bathochromic shift) and lower values (hypochromic shift) and the appearance of new bands for complexes has resulted in the release of three ligands' complex actions towards metal ions. Complexes also present bands within the range 410–480 nm which can be due to the transition of ligand–metal charges for three ligands [34, 36]. The molar absorptivity (ε) values of the prepared metal complexes under investigation were determined (Additional file 1: Table S1) using the relation: A = εcl, where, A = absorbance, c = 1.0 × 10^–3^ M, l = length of cell (1 cm) [22]. The values of 10Dq (difference between t_2g_ and e_g_) for the complexes were calculated by using the following Eq. 10Dq = E = hcν^−^ where E = energy, h = blank constant = 6.626 × 10^−34^ J.sec, c = 3 × 10^10^ cm/sec, ν^−^ = wave number cm^−1^ the data listed in Additional file 1: Table S1.

#### The ^1^H NMR spectra

**Fig. 3:**
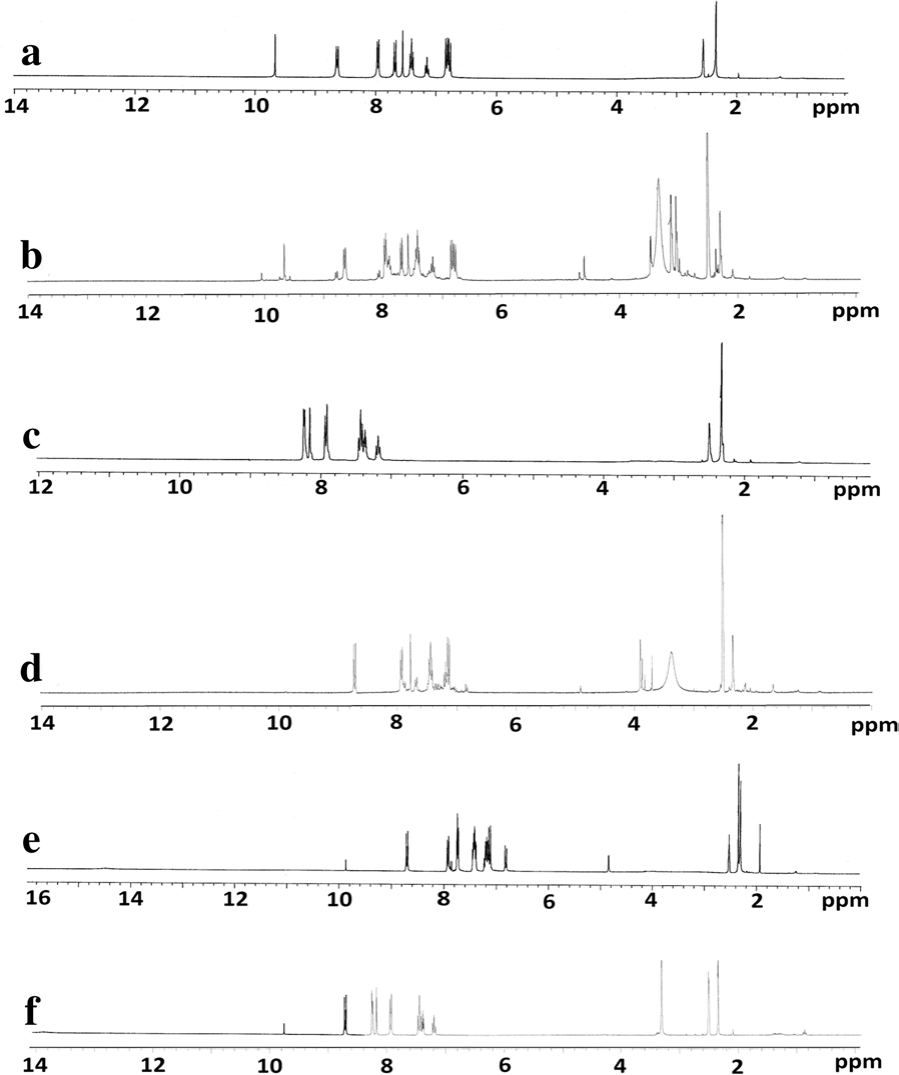
^1^H NMR spectra for **a** L_1_, **b** [Ag(L_1_)_2_(H_2_O)_2_]NO_3_, **c** L_2_, **d** [Ag(L_2_)_2_(H_2_O)_2_]NO_3_.H_2_O, **e** L_3_ and **f** [Ag(L_3_)_2_(H_2_O)_2_]NO_3_

Suggested structure of the isolated Ag(I) complexes confirm about the efficiency of ^1^H NMR spectra. Compared to the one of their complexes (Additional file 1: Table S2), the ^1^H NMR spectra of new free three ligands in DMSO-d_6_. The ^1^H NMR spectra of L_1_ and its metal complex shown in (Fig. 3a, b), the proton of (= CH-Ar) group observed in δ: 9.66 ppm and the protons of aromatic ring of (s, 9H, Aromatic–H) observed at δ: 7.14–7.97 ppm also the values of protons of -CH aliphatic observed in the range δ: 3.03–3.33 ppm (s, 6H, –N (CH_3_)_2_), the proton of (s, 3H, –CH_3_) group observed in δ 2.28 ppm, no major differences were observed as opposed to the Ag(I) complex except that the signal is observed in 3.46 ppm due to H_2_O molecules [36]. This supports the hypothesis that L_1_ interacts as a monodentate ligand bound to the Ag(I) ion through the hydrazono nitrogen group. [37]. The ^1^H NMR spectra of L_2_ and its Ag(I) complex shown in (Fig. 3c, d), the proton of (= CH-Ar) group observed in δ: 8.25 ppm and, the protons of aromatic ring of (s, 8H, Aromatic–H) observed at δ: 7.39–7.91 ppm [38]. The proton of (s, 3H, –CH_3_) group observed in δ 2.30 ppm, simple differences are shown in comparison to the metal complex and the signal is observed in π: 3.47 ppm due to H_2_O molecules. This reinforces the hypothesis that L_2_ reacts via the hydrazono nitrogen group as a monodentate ligand bound to the Ag(I) ion. The ^1^H NMR spectra of L_3_ and its Ag(I) complex shown in (Fig. 3 (E, F)), the proton of (= CH-Ar) group observed in δ: 8.71 ppm and the protons of aromatic ring of (s, 9H, Aromatic–H) observed at δ: 7.18–7.46 ppm also the values of protons of -CH aliphatic observed in the range δ: 3.31 ppm (s, 3H, –O-CH_3_), the proton of (s, 3H, –CH_3_) group observed in δ 2.33 ppm, no major variations were noticed as opposed to the Ag(I) series. This supports the assumption that L_3_ reacts as a monodentate ligand bound to the Ag(I) ion via the hydrazone nitrogen group.

#### Thermal studies

The thermal degradation of ligand (L_1_) began at 190 °C and decay occurs at various temperatures at 310, 544 °C at one stage (Additional file 1: Fig. S1a). This step is accompanied by a net weight loss of 92.36 percent, equivalent to the predicted 92.07 percent. Corresponding to the loss of 8C_2_H_2_ + NH_3_ + CO + N_2_ molecule and 95.65KJ mol^−1^ (endothermic) activation energy. The residue value decomposes at a height of 800 °C and the actual losing weight at this point is 7.64 percent, close to the estimated 7.86 percent equal to 2C. The [Ag(L_1_)_2_(H_2_O)_2_]NO_3_ complex decomposed in two steps (Additional file 1: Fig. S1b), The first one begins at a limit of 189 °C and is followed by a 33.378 percent weight loss leading to a 9C_2_H_2_ + 2H_2_O loss similar to the estimated value of 33.062 percent with an activation energy of 34.37 kJ mol^−1^. The second step occurs at 366 and 562 °C followed by a weight loss of 52.79 percent; equivalent to a value of 8C_2_H_2_ + 4HCN + NO + 2N_2_O, potentially similar to the measured value of 53.798 percent. The residue value proceeds at 931 °C and the overall weight loss from this stage is 13.474 percent, referring to Ag, similar to the 13.14 percent estimated value (Table 2).

**Table 2:** Thermogravimetric data of L_1_, L_2_,L_3_ and their metal complexes

Compounds	Decomposition	DTG _max_ (^◦^C)	% Estimated (calculated)		Assignment
			Mass loss	Total mass loss	Lost species
L_1_	First step	190,310,544,800	92.36 (92.07)	92.36 (92.07)	8C_2_H_2_ + CO + NH_3_ + N_2_
305.19, C_19_H_19_ON_3_	Residue		7.64 (7.86)		2C
[Ag(L_1_)_2_(H_2_O)_2_]NO_3_	1st step	189	33.378 (33.062)	86.526 (86. 86)	9C_2_H_2_ + 2H_2_O
816.65, AgC_38_H_42_N_7_O_7_	Second step	366,562,	52.79 (53.798)		8C_2_H_2_ + 4HCN + NO + 2NO_2_
	Residue	807,931	13.474 (13.14)		Ag
L_2_	First step	273,475,771	86.70(86.56)	86.7(86.56)	6C_2_H_2_ + + SO + N_2_
268, C_15_H_12_N_2_OS	Residue		13.30(13.43)		3C
[Ag(L_2_)_2_(H_2_O)_2_]NO_3_.H_2_O	First step	99	2.08 (2.37)	75. 90 (76.404)	H_2_O
760.59, AgC_30_H_30_N_5_O_8_S_2_	Second step	203,528	73.82 (74.034)		10C_2_H_2_ + 4HCN + 2H_2_O + NO_2_ + SO + SO_2_
	Residue	881	23.35 (23.596)		Ag + 6C
L_3_	First step	120, 291	70.55(71.23)	99.834 (99.99)	8C_2_H_2_
292, C_18_H_16_O_2_N_2_	Second step	518	28.604 (28.76)		2CO + N_2_
[Ag(L_3_)_2_(H_2_O)_2_]NO_3_	First step	244	51.071 (50.60)	82.241 (81.85)	14C_2_H_2_ + 2H_2_O
790.57, AgC_36_H_36_N_5_O_9_	Second step	534	31.17 (31.25)		C_2_H_2_ + CO + 2HCN + 3NO_2_
	Residue	677	17.76 (18.15)		Ag + 3C

The ligand (L_2_) degradates at 273, 475 °C. This stage is followed by a complete loss of weight of 86.70 percent, close to 86.56 percent of the estimated value (Additional file 1: Fig. S1c). Equivalent to 6C_2_H_2_ + SO + N_2_ loss and 31.93 kJ mol^−1^ (endothermic) activation energy. Decomposition of the residual value occurs at 771 °C and the real weight loss from this stage is 13.30 percent, similar to the estimated value of 13.43 percent corresponding to 3C. The [Ag(L_2_)_2_(H_2_O)_2_]NO_3_.H_2_O complex decomposes at two levels of decay (Additional file 1: Fig. S1d), the first phase occurs at 99 °C and is followed by a weight loss of 2.08 per cent relating to the removal of H_2_O, activation energy of 79.28 kJ mol^−1^. The second step of decomposition occurs at temperature is 203, 528 and is accompanied by a weight loss of 75.90%; corresponding to the value of 10C_2_H_2_ + 4HCN + 2H_2_O + NO_2_ + SO + SO_2_ theoretically, close to the calculated value 76.404%. The Residue value decomposition occurs at maximum 881 °C and the actual weight loss from this step is 23.35%, corresponding to Ag + 6C, close to the calculated value 23.596%.

The thermal decay of L_3_ happens in two phases of degradation (Additional file 1: Fig. S1e), the first step arises at 291 °C and is followed by a weight loss of 70.55 percent leading to a loss of 8C_2_H_2_ similar to the measured value of 71.23 per cent with activation energy of 35.31 kJ mol^−1^. The second step occurs at 518 ^ο^C and is accompanied by a weight loss of 28.604%; corresponding to the value of 2CO + N_2_ theoretically, close to the calculated value 28.67%. The [Ag(L_3_)_2_(H_2_O)_2_]NO_3_ degradation takes place in two stages (Additional file 1: Fig. S1f), the first occurs at 244 ^ο^C and is accompained by a weight loss of 51.071% corresponding to loss of 14C_2_H_2_ + 2H_2_O close to the calculated value 50.60% with an activation energy 15.31 kJ mol^−1^. The second one begins at 543 ^ο^C and is followed by a weight loss of 30.17%; corresponding to C_2_H_2_ + CO + 2HCN + 3NO_2_ theoretically, close to the calculated value 31.25%. The Residue remains at 677 °C and the actual weight loss is 17.76%, equal to Ag + 3C, close to the calculated value 18.15%.

#### Kinetic data

The kinetic parameters (activation energy, E*, entropy, ΔS*, enthalpy, ΔH*, and Gibbs free energy, ΔG*) have been evaluated by using the two mentioned methods in the literature [39, 40] and shown in Additional file 1: Fig. S2 and listed in Table 3. The correlation coefficient for Arrhenius plots of thermal degradation stages were found to be in the range 0.943–0.985, revealing a good fit with linear function. The activation energies of decomposition were observed to be in the range 7.44–154.69 kJ mol^−1^. The negative values of ΔS* indicate that the activation complex has a more ordered structure than the reactants or the reactions are slow. The positive ΔH* values postulate an endothermic nature of the formed complexes. The greater positive values of E* indicate that the processes involving in translational, rotational, vibrational states and a changes in mechanical potential energy for complexes and reflect the thermal stability of the complexes [41].

**Table 3:** Thermal behavior and kinetic parameters determined using the Coats–Redfern (CR) and Horowitz–Metzger (HM) operated for L_1,_ L_2_, L_3_ and their complexes

Compounds	Decompositi range(K)	T_s_(K)	Method	Parameter					R^a^	SD^b^
				E* (kJ mol^−1^)	*A* (s^−1^)	ΔS* (J mol^−1^ K^−1^)	ΔH* (kJ mol^−1^)	ΔG* (kJ mol^−1)^		
L_1_	673–905	817	CR HM	95.656 116.813	69.368 × 10^3^ 30.233 × 10^3^	− 432.844 − 425.910	88.863 110.020	442.497 457.988	0.970 0.984	0.187 0.065
[Ag(L_1_)_2_(H_2_O)_2_]NO_3_	373–573	462	CR HM	34.379 38.825	3.390 × 10^2^ 2.621 × 10^3^	− 393.344 − 410.348	30.537 34.983	212.262 224.564	0.984 0.970	0.116 0.087
L_2_	453–645	546	CR HM	31.931 40.983	37.451 672.979	− 373.640 − 397.656	27.391 36.443	231.398 253.563	0.943 0.945	0.207 0.112
[Ag(L_2_)_2_(H_2_O)_2_]NO_3_.H_2_O	689–881	801	CR HM	79.284 93.856	6.618 × 10^3^ 113.45 × 10^3^	− 413.475 − 437.099	72.624 87.196	403.817 437.312	0.945 0.940	0.220 0.116
L_3_	438–630	564	CR HM	35.317 48.603	1.185 × 10^2^ 2.847 × 10^3^	− 382.948 − 409.379	30.627 43.913	246.610 274.803	0.960 0.952	0.182 0.106
[Ag(L_3_)_2_(H_2_O)_2_]NO_3_	365–685	497	CR HM	15.316 22.370	0.758 0.119 × 10^2^	− 342.002 − 364.917	11.183 18.237	181.158 199.601	0.981 0.985	0.089 0.054

^a^Correlation coefficients of the Arrhenius plots and ^b^Standard deviation

#### Mass spectra

**Fig. 4:**
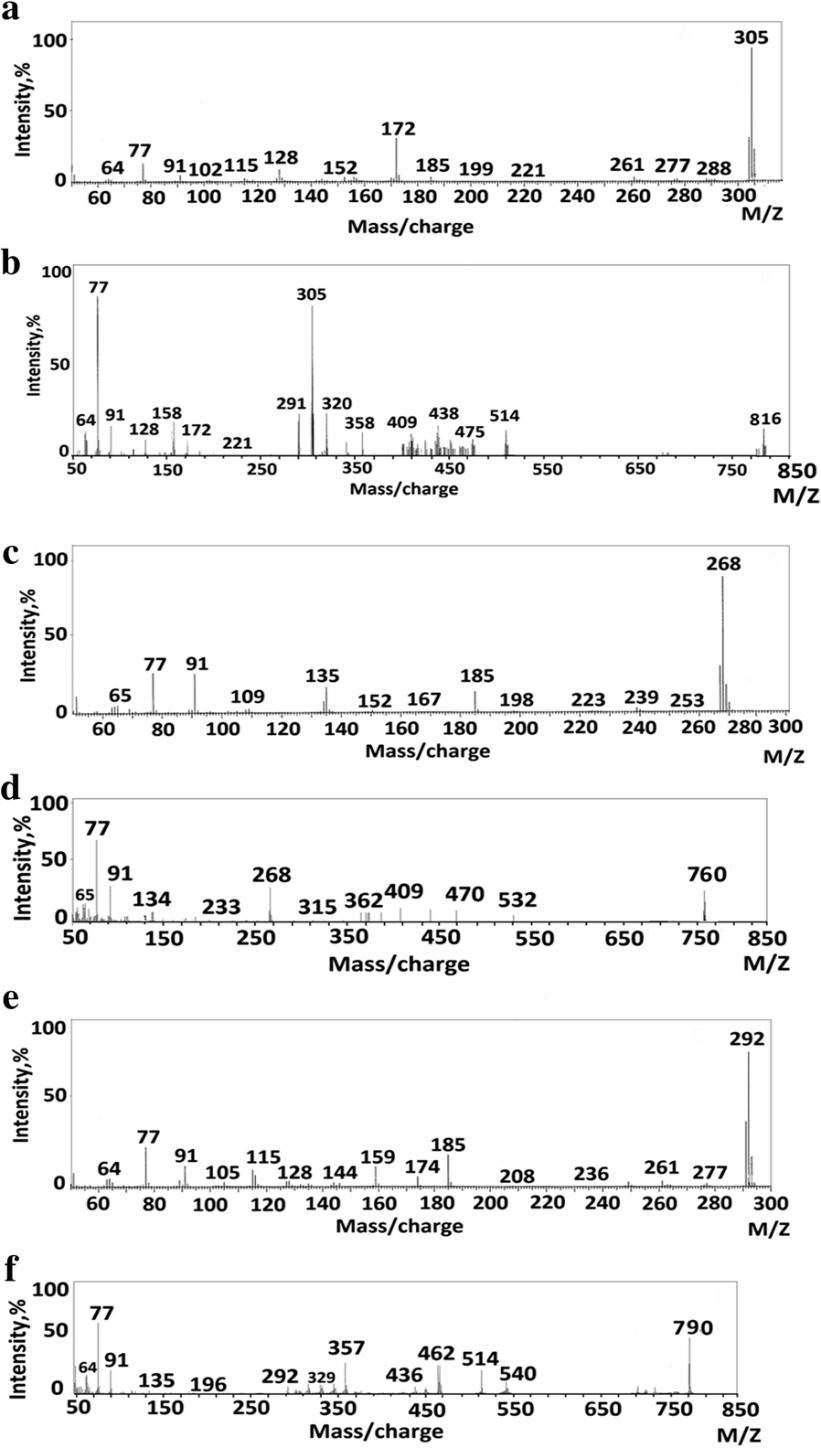
Mass spectra diagrams for (A) L_1_, (B) [Ag(L_1_)_2_(H_2_O)_2_]NO_3_, (C), L_2_ (D) [Ag(L_2_)_2_(H_2_O)_2_]NO_3_.H_2_O, (E) L_3_ and (F) [Ag(L_3_)_2_(H_2_O)_2_]NO_3_

**Scheme 3:**
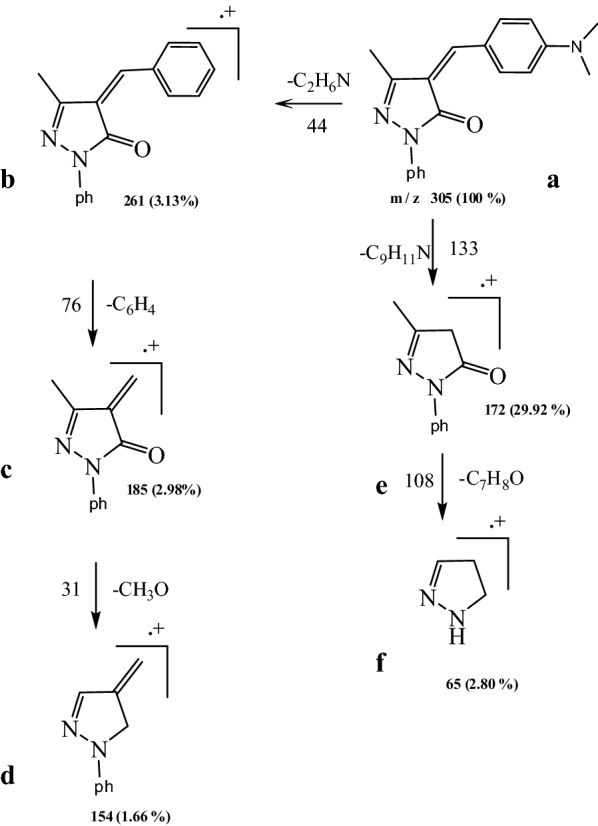
Fragmentation pattern of L_1_

**Scheme 4:**
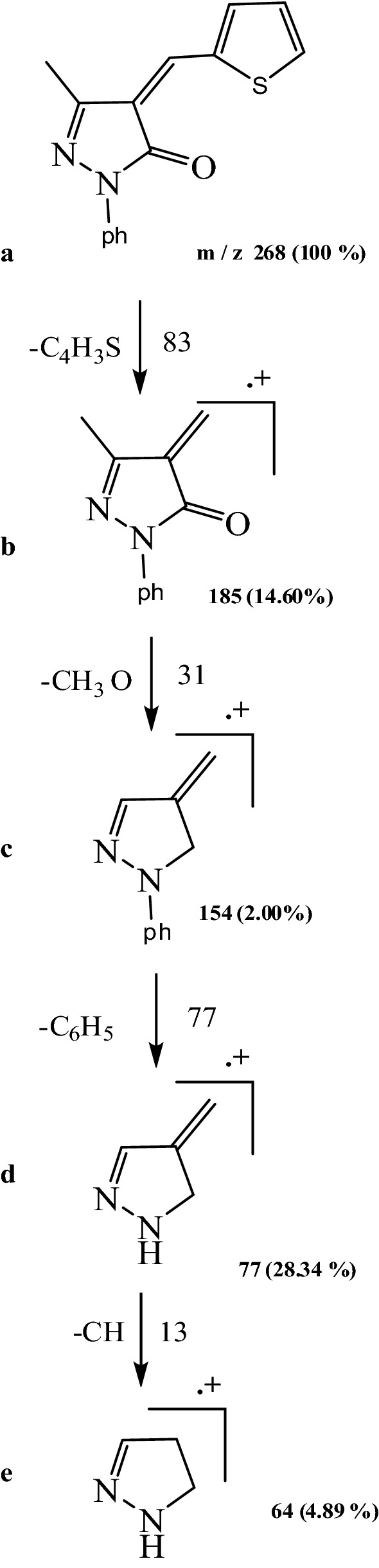
Fragmentation pattern of L_2_

**Scheme 5:**
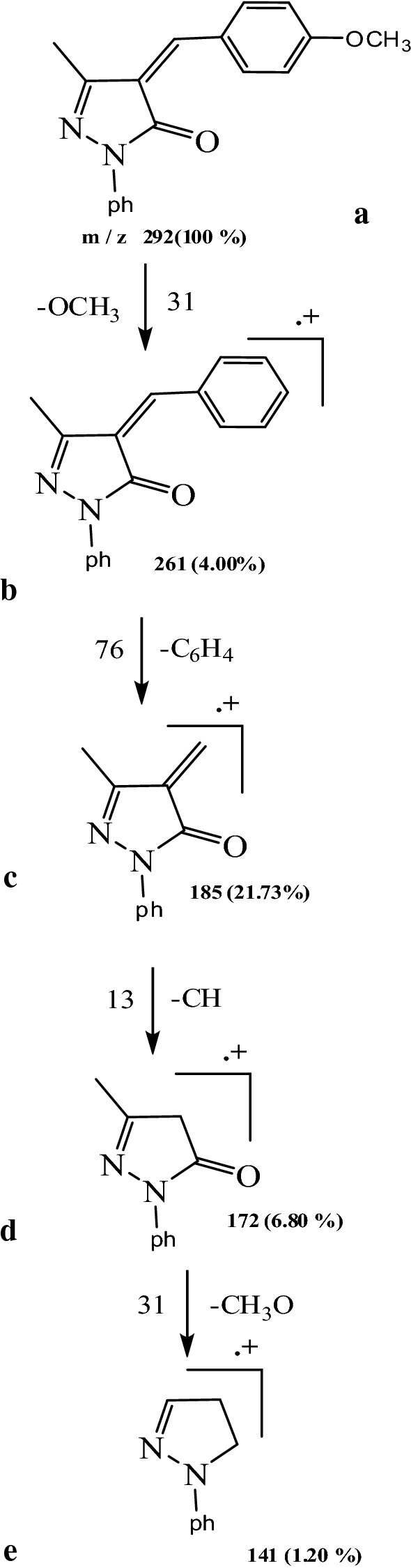
Fragmentation pattern of L_3_

The principle of a mass spectrometer focuses on the separation of fragments of ions based on the distribution of these ions with the mass to charge ratio (m/z). The L_1_, L_2_, L_3_ fragmentation patterns and their complexes were obtained from the mass spectra, and were in good agreement with the structure suggested. The L_1_ showed molecular ion peak (M^+.^) with m/z = 305 (100%). The molecular ion peak [a] losses C_2_H_6_N to give fragment [b] at m/z = 261 (3.13%), then [b] losses C_6_H_4_ to give fragment [c] at m/z = 185 (2.98%) and [c] losses CH_3_O to give [d] at m/z = 154(0.66%). The molecular ion peak [a] losses C_9_H_11_N to give fragment [e] at m/z = 172 (29.92%) and this [e] losses C_7_H_8_O to give fragment [f] at m/z = 64 (2.08%) (Fig. 4), (Scheme 3). Fragmentation pattern of the complex [Ag(L_1_)_2_(H_2_O)_2_]NO_3_ is given as an example in (Fig. 4), Additional file 1: Scheme S1. The molecular ion peak [a] appeared at m/z = 816 (20.5%) losses C_18_H_22_N_2_ to give [b] at m/z = 514 (17.7%) and it losses C_2_H_6_O_2_ to give [c] at m/z = 452 (11.7%). The L_2_ molecular ion peak [a] appeared at m/z = 268 (100%) losses C_4_H_3_S to give [b] at m/z = 185 (14.60%) then it losses CH_3_O to give [c] at m/z = 154 (0.2%), molecular ion peak[c] lossC_6_H_5_ to give [d] at m/z = 77(28.34%) and molecular ion peak [d] losses CH to give [e] at m/z = 64 (4.89%).(Fig. 4), Scheme 4. Fragmentation pattern of the complex [Ag(L_2_)_2_(H_2_O)_2_]NO_3_.H_2_O is given as an example in (Fig. 4), Additional file 1: Scheme S2. The molecular ion peak [a] appeared at m/z = 760 (35%) losses C_10_H_8_S_2_to give [b] at m/z = 532 (5%) and it losses C_2_H_6_O_2_ to give [c] at m/z = 470 (12%). The L_3_ molecular ion peak [a] appeared at m/z = 292 (100%) losses CH_3_O to give [b] at m/z = 261 (4%) then [a] losses C_6_H_4_ to give [c] at m/z = 185 (21.73%), molecular ion peak[c] loss CH to give [d] at m/z = 172(6.8%) and molecular ion peak [d] losses CH_3_O to give [e] at m/z = 141 (1.2%).(Fig. 4), Scheme 5. Fragmentation pattern of the complex [Ag(L_3_)_2_(H_2_O)_2_]NO_3_ is given as an example in (Fig. 4), Additional file 1: Scheme S3. The molecular ion peak [a] appeared at m/z = 790 (65%) losses C_2_H_6_O_2_to give [b] at m/z = 692 (2%), it losses C_12_H_8_ to give [c] at m/z = 540 (12.5%) and molecular ion peak [c] losses C_2_H_2_ to give [d] at m/z = 514 (25.3%) [42].

### Biological activity studies

#### Antimicrobial studies

**Fig. 5:**
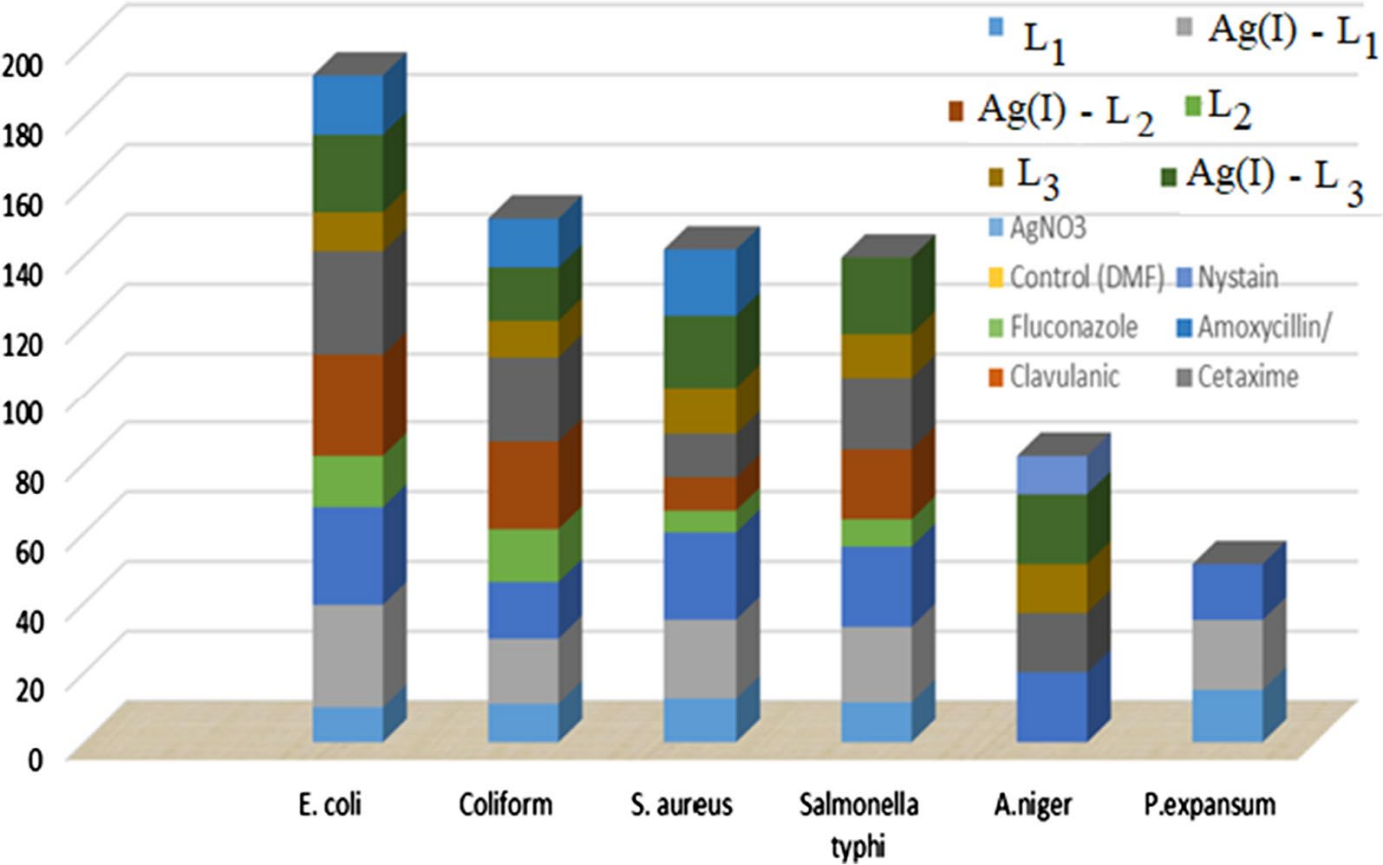
Statistical representation for biological activity of L_1_, L_2_, L_3_ and its metal complexes

The antimicrobial efficacy of L_1_, L_2_, L_3_ and their free ligand complexes are explored in this experiment. Studies were conducted on *E. Coli* ATCC11229, *Coliform* ATCC8729, *S. aureus* ATCC6538, and *Salmonella typhi* ATCC14028 and fungal species as *A. niger* and *P. expansum* screening was tested against and examination and evaluation of the prepared complexes [42]. The same results were reported for *E. Coli* ATCC11229 of Ag(I)-L_2_ and Ag (I)-L_1_ followed by Ag(I)-L_3_ considers that the lowest findings are equivalent to those of other complexes. The effect of free ligands on this strain has been shown to be below its complex and can be organized according to the sensitivity of the strains L_2_, L_3_ and L_1_ in the following ascending order. The effect of Ligands and their complexes on *Coliform* ATCC8729 showed that Ag(I)-L_2_ is highly important, giving 25.12 mm respectively. Although the remaining complexes showed lower results than the L_2_ complexes. The results obtained in Table 4 and Fig. 5 showed that lower activity on the same strain and these results ensured that free ligand complexes were more active than free ligand complexes. In gram + ve bacteria, *S. aureus* ATCC6538, Highly important antibacterial activity of metal complexes with L_1_ followed L_3_ complex. The lesser activity from ligand L_2_ and its complex. The antibacterial activity of metal complexes on *Salmonella typhi* ATCC14028 showed a good activity against (gram −ve), that recorded the best results Ag(I)-L_3_ > Ag(I)-L_1_ > Ag(I)-L_2_ respectively. The action of the free ligands on gram –ve bacteria has yielded results lower than their complexes which give respectively 12.6, 11.43 and 7.8 mm, L_3_, L_1_, L_2_. The presence of different ligands and other complexes on both fungal strains of the testes, *A. niger* recorded that Ag(I)-L_3_ showed a significant difference the highly results (20 ± 2.6) though free L_3_ results showed less than its complex. Others did not show any activity against tested fungi (*A. niger*). The effect of various significant ligands and other complexes on *P. expansum* did not show any activity whereas the the highest broad spectrum of activity on the same test strain showed the best results on L_1_ and its complexes [42].

**Table 4:** The inhibitation diameters zone values (mm) for L_1,_ L_2_, L_3_ and its complexes

Compounds		Microbial species					
		Bacteria				Fungi	
		*E. coli*	*Coliform*	*S. aureus*	*Salmonella typhi*	*A.niger*	*P.expansum*
L_1_		10^+1^ ± 1.1	11^+1^ ± 1.8	12.5^+1^ ± 0.89	11.43^+3^ ± 0.79	^NA^	20^+3^ ± 1.3
L_1_ / Ag(I)		29.33^+2^ ± 1.58	18.65^+2^ ± 1.45	22.6^+1^ ± 1.88	21.6^+3^ ± 1.98	^NA^	16^+3^ ± 0.75
L_2_		14.66^+1^ ± 1.1	15.13^+1^ ± 1.9	6.25^+1^ ± 0.81	7.8^+1^ ± 0.54	^NA^	^NA^
L_2_ / Ag(I)		29.6^+2^ ± 1.75	25.12^+3^ ± 1.33	9.5^+1^ ± 0.74	20^+3^ ± 2.1	^NA^	^NA^
L_3_		11.2^+1^ ± 1.4	10.5^+1^ ± 0.95	13.6^+1^ ± 1.3	12.6^+3^ ± 0.78	14^+2^ ± 0.75	^NA^
L_3_ / Ag(I)		22.16^+1^ ± 2.4	15.32^+1^ ± 1.3	20.8^+1^ ± 2.2	22^+3^ ± 2.2	20^+3^ ± 2.6	^NA^
AgNO_3_			–	–	–	–	–
Control (DMF)		–	–	–	–	–	–
Standard	Nystain	–	–	–	–	11 ± 1.1	00
	Fluconazole	–	–	–	–	00	00
	Amoxycillin/Clavulanic	17 ± 1.1	14 ± 1.3	19 ± 1.8	00	–	–
	Cetaxime	00	00	00	00	–	–

Statistical significance P^NS^ – P not significant, *P* > 0.05; *P*^+1^ – P significant, *P* < 0.05; P^+2^ – P highly significant, *P* < 0.01; P^+3^ – P very highly significant, *P* > 0.001; Student’s* t*-test (Paired)

Normal antibiotic efficacy of antimicrobials (AMC, CTX, NS, FU). The AMC mixture give the effective against *E. coli*, *Coliform*, *S. aureus* and NS high inhibitory activity on *A. niger*. Other antibiotics have shown no action on other microorganisms. Eventually, the bacterial strains showed a varied response to the three free ligands and their complex antimicrobial activity, but the results indicated that the high activity of ligand complexes was better than their free ligands. The two fungal strains are more resistant to synthesis ligands and their complexes than bacterial strains [42–46].

#### Determination of MIC for the most sensitive organisms

**Fig. 6:**
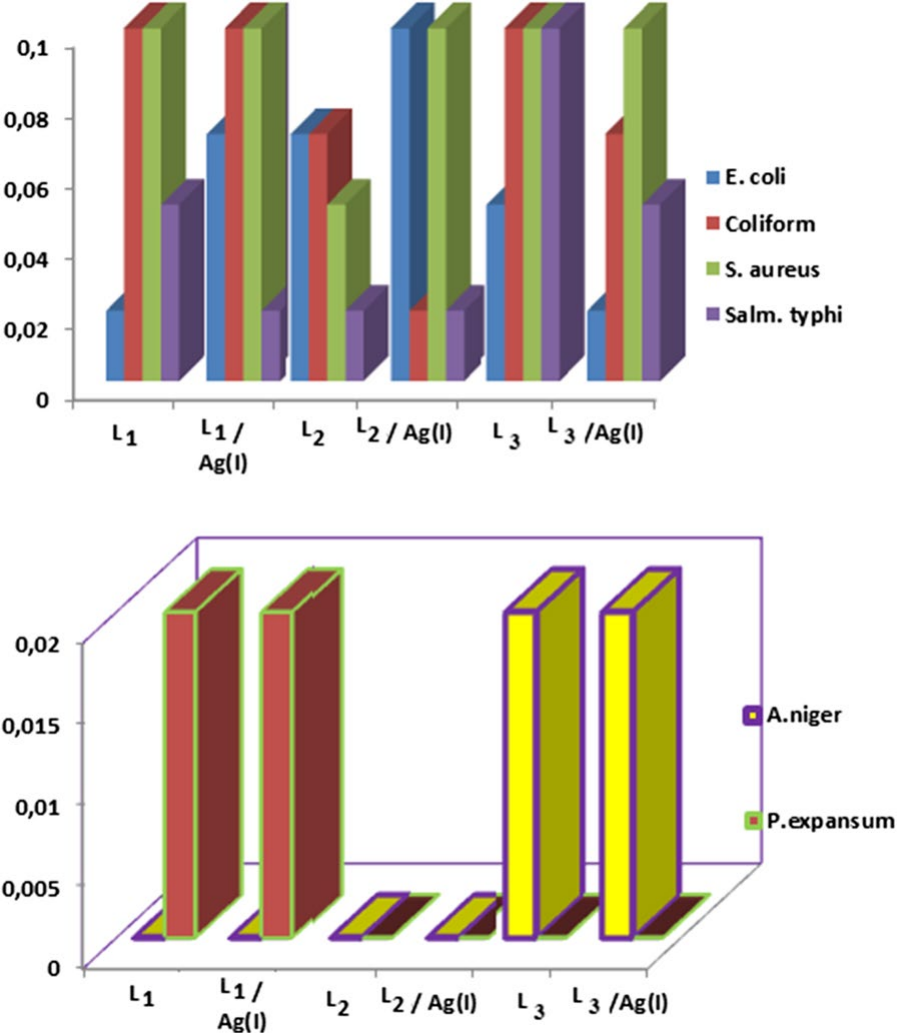
MIC for the most sensitive organisms

The artificial ligands and their complexes developed the biological efficacy towaeds the more resistant bacteria and fungi (Table 5A–D) and Fig. 6). The order of The lowest MIC for in case of *E. coli* decrease in order: L_1_ = Ag (I) – L_3_ (0.02 mg/100 mL)˃ L_3_ (0.05 mg/100 mL)˃ Ag (I) – L_1_ = L_2_ (0.07 mg/100 mL) ˃ Ag (I) – L_2_ (0.1 mg / 100 mL) [42], *Coliform* decrease in order: Ag (I) – L_2_ (0.02 mg/100 mL) ˃ Ag (I) – L_3_ = L_2_ (0.07 mg/100 mL) ˃ L_1_ = Ag (I) – L_1_ = L_3_ (0.1 mg/100 mL). *Salmonella typhi* showed that, the amazing results of ligands and its complexes: L_2_, Ag(I) – L_2_ = Ag(I) – L_1_ (0.02 mg/100 mL) ˃ Ag (I) – L_3_ = L_1_ ( 0.05 mg/ 100 mL) ˃ L_3_ (0.1 mg/ 100 mL), *S. aureus* order: L_3_ = Ag(I) – L_2_ = Ag(I) – L_1_ = L_1_ = Ag(I) –L_3_ (0.1 mg/ 100 mL) ˃ L_2_ (0.05 mg/ 100 mL). Table 5E, F and Fig. 6 data showed that the lowest MIC for the two strains measured at conc. 0.02 mg/100 mL. Although MIC at complex L_3_ was recorded by *A.niger*, the same result was recorded on Ag(I)—L_3_ at conc. 0.02 mg/100 mL. Ligand L_1_ and its complexes demonstrate the strongest MIC on *P. expansum*, although no behavior is displayed by the other compounds and their complexes. These findings ensured that the activity of synthetic ligands and their complexes on pathogenic bacteria and fungi demonstrated a minimum inhibitor concentration (MIC) for the most vulnerable pathogens. [42, 47, 48].

**Table 5:** (A) Of One-way ANOVA: *E. coli* vs MIC Compounds. (B) Of One-way ANOVA: Coliform versus MIC Compounds. (C) Of One-way ANOVA: *S. aureus* vs MIC Compounds. (D) Of One-way ANOVA: *Salm. typhi *vs MIC Compounds. (E) Of One-way ANOVA: *A. niger* vs MIC Compounds. (E) Of One-way ANOVA: *A. niger* vs MIC Compounds. (F) Of One-way ANOVA: *P.expansum* vs MIC Compounds

(A)			
Grouping information using the fisher LSD method			
Compounds	N	Mean	Grouping
L_1_	3	0.02	A
L_3_/Ag(I)	3	0.02	A
L_3_	3	0.05	B
L_1_/Ag(I)	3	0.07	B
L_2_	3	0.07	B
L_2_/Ag(I)	3	0.10	C

(B)			
Grouping information using the fisher LSD method			
Compounds	N	Mean	Grouping
L_2_/Ag(I)	3	0.02	A
L_2_	3	0.07	C
L_3_/Ag(I)	3	0.07	C
L_1_	3	0.10	D
L_1_/Ag(I)	3	0.10	D
L_3_	3	0.10	D

(C)			
Grouping information using the fisher LSD method			
Compounds	N	Mean	Grouping
L_3_/Ag(I)	3	0.05	A
L_3_	3	0.05	A
L_2_/Ag(I)	3	0.07	B
L_1_/Ag(I)	3	0.10	C
L_1_	3	0.10	C
L_2_	3	0.10	C

(D)			
Grouping information using the fisher LSD method			
Compounds	N	Mean	Grouping
L_1_/Ag(I)	3	0.02	A
L_2_	3	0.02	A
L_2_/Ag(I)	3	0.02	A
L_1_	3	0.05	B
L_3_/Ag(I)	3	0.05	B
L_3_	3	0.10	C

(E)			
Grouping information using the fisher LSD method			
Compounds	N	Mean	Grouping
L_3_/Ag(I)	3	0.02	A
L_3_	3	0.02	A
L_2_/Ag(I)	3	0.0	B
L_2_	3	0.0	B
L_1_/Ag(I)	3	0.0	B
L_1_	3	0.0	B

(F)			
Grouping information using the fisher LSD method			
Compounds	N	Mean	Grouping
L_1_/Ag(I)	3	0.02	A
L_1_	3	0.02	A
L_3_/Ag(I)	3	0.0	B
L_3_	3	0.0	B
L_2_/Ag(I)	3	0.0	B
L_2_	3	0.0	B

Means that do not share a letter are significantly different

Fisher 95% Simultaneous Confidence Intervals

## Conclusion

Development and characterisation of three novel complexes of some replaced pyrazole derivatives as ligands (4-(4-dimethylamino benzylidene)-3-methyl-1-phenyl-1*H*-pyrazol-5(4*H*)-one (2a) L_1_, 4-(4-Thiophene)-3-methyl-1-phenyl-1*H*pyrazol-5(4*H*)-one (2b) L_2_, 4-(4-methoxy benzylidene)-3-methyl-1-phenyl-1*H*pyrazol-5(4*H*)-one (2c) L_3_) with Ag(I) was achieved using physicochemical and spectroscopic methods.. In the resulting complexes, L_1_, L_2_, and L_3_ were bound by the nitrogen atom to the metal ion via ν(C = N). For the three ligands and their complexes, thermogravimetric kinetic parameters and their differential were evaluated using the Coats-Redfern and Horowitz-Metzger equations. Metal complexes exhibited higher inhibition against all tested microorganisms and pathogenic bacteria and fungi and were the most susceptible pathogens with a minimum inhibitory concentration ( MIC).

## Methods

### Chemistry

Analytical grade reagents, commercially available from multiple suppliers and used without further purification, were all the chemicals used in the complex preparation. Synthesized compounds and their complexes have been characterized by elemental analysis, magnetic and spectroscopic methods (IR, ^13^C, ^1^HNMR, mass) and thermal analysis using the known apparatuses [42].

### Synthesis of the ligands

#### Common 3-methyl-1-phenyl-5-pyrazolone synthesis technique (1)

Pure ethyl acetoacetate (0.05 mol, 6.2 mL) was mixed with pure phenyl hydrazine (0.05 mol, 5 mL), 0.5 mL of acetic acid was added, according to knowm method [42]. Methyl phenyl pyrazolone was obtained as colorless crystals, 127 °C melting point and 83.6 percent yield [27].

#### Specific method for preparing derivatives of 4-arylidene-3-methyl-1-phenyl-5-pyrazolone (2a-c)

The oil bath heated a mixture of 1-aryl-3-methyl-5-pyrazolone (0.01 mol, 1.74 g) and replaced aromatic aldehydes (0.012 mol) at 150–160 °C for 2-4hrs. TLC has tracked the progress of the reaction using ethyl acetate: hexane (9:1) as solvent. The mixture was cooled, triturated and washed off with ether (20 mL). The colored residue was recrystallized from ethanol to provide the corresponding 4-arylidene-3-methyl-1-phenyl-5-pyrazolone (2a-c) as colored products, respectively [28].

4-(4-dimethylamino benzylidene)-3-methyl-1-phenyl-1*H*-pyrazol-5(4*H*)-one (2a) L_1_.

4-(4-Thiophene)-3-methyl-1-phenyl-1*H*pyrazol-5(4*H*)-one (2b) L_2_.

4-(4-methoxy benzylidene)-3-methyl-1-phenyl-1*H*pyrazol-5(4*H*)-one (2c) L_3_.

### 4-(4-dimethylamino benzylidene)-3-methyl-1-phenyl-1*H*-pyrazol-5(4*H*)-one (2a) L_1_

Brick Red, mp = 170 °C, yield 83% IR (KBr, *v*, cm^−1^): 3444 (OH), 1670 (C = O), and 1550 cm^−1. 1^H NMR (DMSO-*d*_6_, 300 MHz): δ = 2.28 (s, 3H, CH_3_), 3.03 (s, 6H, -N (CH_3_)_2_), 7.14 (S, 1H, = CH-Ar), 9.66 (d, 3H, Ar–H),. Anal. Calcd for C_19_H_19_N_3_O (305.19): C, 74.40; H, 6.22; N 13.76; Found C, 74.23; H, 6.13; N, 13.35%.

### 4-(4-Thiophene)-3-methyl-1-phenyl-1*H*pyrazol-5(4*H*)-one (2b) L_2_

Orange, mp = 125 °C, yield 74% IR (KBr, *v*, cm^−1^): 3448 (OH), 1681 (C = O), 1496 cm^−1^ (C = N) and 1056 cm^−1^(C = S). ^1^H NMR (DMSO-*d*_6_, 300 MHz): δ = 2.30 (s, 3H, CH_3_), 7.39 (S, 1H, = CH-Ar), 8.25 (d, 3H, Ar–H). Anal. Calcd for C_15_H_12_N_2_OS (268): C, 67.16; H, 4.47; N 10.44; S, 11.94; Found C, 67.00; H, 4.32; N, 10.21; S, 11.65%.

### 4-(4-methoxy benzylidene)-3-methyl-1-phenyl-1*H*pyrazol-5(4*H*)-one (2c) L_3_

Orange, mp = 122 °C, yield 82% IR (KBr, *v*, cm^−1^): 3444 (OH), 1678 (C = O), 1508 cm^−1^ (C = N) and. ^1^H NMR (DMSO-*d*_6_, 300 MHz): δ = 1.91 (s, 3H, CH_3_), 3.69 (s, 3H, -OCH_3_), 7.20 (S, 1H, = CH-Ar), 8.71 (d, 3H, Ar–H).Anal. Calcd for C_18_H_16_N_2_O_2_ (292): C, 73.97; H, 5.47; N 9.58; Found C, 73.78; H, 5.13; N, 9.34%.

### Synthesis of the complexes

The brown solid complex [Ag(L_1_)_2_(H_2_O)_2_]NO_3_ was prepared by adding 0.5 mmol (0.085 g) of AgNO_3_ in 20 ml of acetone to a stirred suspended solution 1 mmol (0.305 g) of L_1_ in 50 ml acetone. The reaction mixture was refluxed for 6 h, the precipitate was drained off, washed several times with acetone and dried under vacuum over anhydrous CaCl_2_. Dark brown [Ag(L_2_)_2_(H_2_O)_2_]NO_3_.H_2_O, [Ag(L_3_)_2_(H_2_O)_2_]NO_3_ solid complexes were prepared in the same manner as mentioned above.

### [Ag(C_19_H_19_N_3_O)_2_(H_2_O)_2_]NO_3_ (AgC_38_H_42_N_7_O_7_) complex

Brown; Yield: 85%; m.p.: 160 ^ο^C; M.Wt: 816.65; Elemental analysis for AgC_38_H_42_N_7_O_7_: found, C, 55.31; H, 4.99; N, 12.00; Ag, 13.14; Calcd, C 55.89; H, 5.18; N, 12.01; Ag, 13.21; Λ_m_ = 115.75 S cm^2^ mol^−1^; IR (KBr, *v*, cm^−1^): 3450 m,br (OH), 1666 m (C = O), 1523vw cm^−1^(C = N) and 813w and 837w (M–N). ^1^H NMR (DMSO-*d*_6_, 300 MHz): δ = 2.49 (s, 3H, CH_3_), 3.46 (s, 2H, H_2_O), 2.27–2.33 (s, 6H, -N (CH_3_)_2_), 9.67 (S, 1H, = CH-Ar), 7.14–7.97 (m, 4H, Ar–H).

### [Ag(C_15_H_12_N_2_OS)_2_(H_2_O)_2_]NO_3_.H_2_O (AgC_30_H_30_N_5_O_8_S_2_) complex

Dark brown; Yield: 74%; m.p.: 125 ^ο^C; M.Wt: 760.59; Elemental analysis for AgC_30_H_30_N_5_O_8_S_2_: found, C, 47.22; H, 3.91; N, 9.15; Ag, 14.13; Calcd, C, 47.37; H, 3.98; N, 9.21; Ag, 14.18; Λ_m_ = 135.50 S cm^2^ mol^−1^; IR (KBr, *v*, cm^−1^): 3444 m, br (OH), 1685 m (C = O), 1527vw cm^−1^ (C = N), 1099 m cm^−1^(C = S), 748w and 792w (M–N). ^1^H NMR (DMSO-*d*_6_, 300 MHz): δ = 2.49 (s, 3H, CH_3_), 3.37 (s, 2H, H_2_O), 8.64 (S, 1H, = CH-Ar), 7.20–7.94 (d, 3H, Ar–H).

### [Ag(C_18_H_16_N_2_O_2_)_2_(H_2_O)_2_]NO_3_ (AgC_36_H_36_N_5_O_9_) complex

Dark brown; Yield: 90%; m.p.: 150 ^ο^C; M.Wt: 790.57; Elemental analysis for AgC_36_H_36_N_5_O_9_: found, C, 54.47; H, 4.11; N, 8.80; Ag, 13.60; Calcd, C, 54.69; H, 4.59; N, 8.86; Ag, 13.64; Λ_m_ = 114.52 S cm^2^ mol^−1^; IR (KBr, *v*, cm^−1^): 3444 (OH), 1678 (C = O), 1520 cm^−1^ (C = N), 759w and 779w (M–N). ^1^H NMR (DMSO-*d*_6_, 300 MHz): δ = 2.33 (s, 3H, CH_3_), 3.31 (s, 3H, -OCH_3_), 8.42 (S, 1H, = CH-Ar), 7.18–7.46 (d, 3H, Ar–H).

## Supplementary Information

**Additional file 1: Table S1. **UV-Vis. spectral data of the free ligand L_1_, L_2_, L_3_ and their Ag(I)-complexes. **Table S2. **Selected ^1^H NMR data of L_1_, L_2_, L_3_ and its diamagnetic complexes. **Fig. S1. **TGA and DTG diagrams for **a** L_1_, **b** [Ag(L_1_)_2_(H_2_O)_2_]NO_3_, **c**, L_2_ **d** [Ag(L_2_)_2_(H_2_O)_2_]NO_3_.H_2_O, **e** L_3_ and **f** [Ag(L_3_)_2_(H_2_O)_2_]NO_3_. **Fig. S2.** The diagrams of kinetic parameters of L_1_, [Ag(L_1_)_2_(H_2_O)_2_]NO_3_, L_2_, [Ag(L_2_)_2_(H_2_O)_2_]NO_3_.H_2_O, L_3_ and [Ag(L_3_)_2_(H_2_O)_2_]NO_3_using Coats-Redfern (CR) and Horowitz-Metzger (HM) equations. **Scheme S1.** Fragmentation pattern of [Ag(L_1_)_2_(H_2_O)_2_]NO_3_. **Scheme S2**. Fragmentation pattern of [Ag(L_2_)_2_(H_2_O)_2_]NO_3_.H_2_O. **Scheme S3.** Fragmentation pattern of [Ag(L_3_)_2_(H_2_O)_2_]NO_3_

## Data Availability

The datasets used and/or analysed during the current study available from the corresponding author on reasonable request.

## Abbreviations

EtOH: Ethanol
NMR: Nuclear magnetic resonance
IR: Infrared radiation
DMSO: Dimethyl sulfoxide
MIC: Minimum inhibation concentrations
