# Supplementary material for: Spectral, thermal, antimicrobial studies for silver(I) complexes of pyrazolone derivatives
Source: BMC Chem. 2020 Dec 5;14(1):69. doi: 10.1186/s13065-020-00723-0 (PMC7719257; doi:10.1186/s13065-020-00723-0)
Supplement: Supplementary file 1 — Additional file 1: Table S1. UV-Vis. spectral data of the free ligand L1, L2, L3 and their Ag(I)-complexes. Table S2. Selected 1H NMR data of L1, L2, L3 and its diamagnetic complexes. Fig. S1. TGA and DTG diagrams for a L1, b [Ag(L1)2(H2O)2]NO3, c, L2 d [Ag(L2)2(H2O)2]NO3.H2O, e L3 and f [Ag(L3)2(H2O)2]NO3. Fig. S2. The diagrams of kinetic parameters of L1, [Ag(L1)2(H2O)2]NO3, L2, [Ag(L2)2(H2O)2]NO3.H2O, L3 and [Ag(L3)2(H2O)2]NO3using Coats-Redfern (CR) and Horowitz-Metzger (HM) equations. Scheme S1. Fragmentation pattern of [Ag(L1)2(H2O)2]NO3. Scheme S2. Fragmentation pattern of [Ag(L2)2(H2O)2]NO3.H2O. Scheme S3. Fragmentation pattern of [Ag(L3)2(H2O)2]NO3 [file 13065_2020_723_MOESM1_ESM.docx]

**Spectral, Thermal, Antimicrobial Studies for silver(I) Complexes of pyrazolone derivatives**

Soha F. Mohamed^1^, Wesam S. Shehab^1^, Aboubakr M. Abdullah^*,2^, Mostafa H. Sliem^2^, Walaa H. El-Shwiniy^*1, 3^

^1^ Department of Chemistry, Faculty of Science, Zagazig University, Zagazig, 44519, Egypt.

^2^Center for Advanced Materials, Qatar University, Doha, P.O. Box 2713, Qatar.

^3^Department of Chemistry, College of Science, University of Bisha, Bisha, 61922, Saudi Arabia.

****Corresponding author, E.mail:*** [bakr@qu.edu.qa](mailto:bakr@qu.edu.qa) ***,*** [abubakr_2@yahoo.com](mailto:abubakr_2@yahoo.com)

**Table S1:** UV-Vis. spectral data of the free ligand L_1_, L_2_, L_3_ and their Ag(I)-complexes.

| Compounds | π-π^*^  transitions  λ_max (nm)_ | ν(cm^-1^) | ϵ  (M^-1^ cm^-1^) | n-π^*^  transitions  λ_max (nm)_ | ν (cm^-1^) | ϵ  (M^-1^ cm^-1^) | Ligand-metal  Charge transfer  λ_max (nm)_ | ν (cm^-1^) | ϵ  (M^-1^ cm^-1^) | d-d  transition  λ_max (nm)_ | ν (cm^-1^) | 10Dq E(J) |
| --- | --- | --- | --- | --- | --- | --- | --- | --- | --- | --- | --- | --- |
| L_1_ | 281 | 35587 | 20 | 330 | 30303 | 160 | - | - | - | - | - | - |
| Ag(I)-L_1_ | 279 | 3584 | 20 | 358 | 27932 | 140 | 420 | 23809 | 10 | - | - | 0 |
| L_2_ | 279 | 3584 | 19 | 330 | 30303 | 145 | - | - |  | - | - | - |
| Ag(I)-L_2_ | 279 | 3584 | 19 | 324 | 30864 | 150 | 410 | 24390 | 13 | - | - | 0 |
| L_3_ | 278 | 35971 | 21 | 330 | 30303 | 130 | - | - | - | - | - | - |
| Ag(I)-L_3_ | 278 | 35971 | 21 | 381 | 26246 | 145 | 480 | 20833 | 15 | - | - | 0 |

**Table S2:** Selected ^1^H NMR data of L_1_, L_2_, L_3_ and its diamagnetic complexes.

| Compounds | *δ* H; H_2_O | *δ*H; -CH aliphatic (methyl) | *δ*H; -CH aromatic | *δ*H; =CH aromatic | *δ*H;-N(CH_3_)_2_ | *δ*H; -OCH_3_ |
| --- | --- | --- | --- | --- | --- | --- |
| L_1_ | 3.421 | 2.288 | 7.147-7.973 | 9.66 | 3.036-3.330 | - |
| L_1_ / Ag (I) | 3.460 | 2.491 | 7.146-7.970 | 9.670 | - | - |
| L_2_ | 3.410 | 2.303 | 7.390-7.917 | 8.250 | - | - |
| L_2_ /Ag(I) | 3.375 | 2.495 | 7.460-7.771 | 8.469 | - | - |
| L_3_ | - | 1.913 | 7.206-7.747 | 8.715 | - | 3.694 |
| L_3_ / Ag (I) | - | 2.339 | 7.189-7.465 | 8.421 | - | 3.321 |


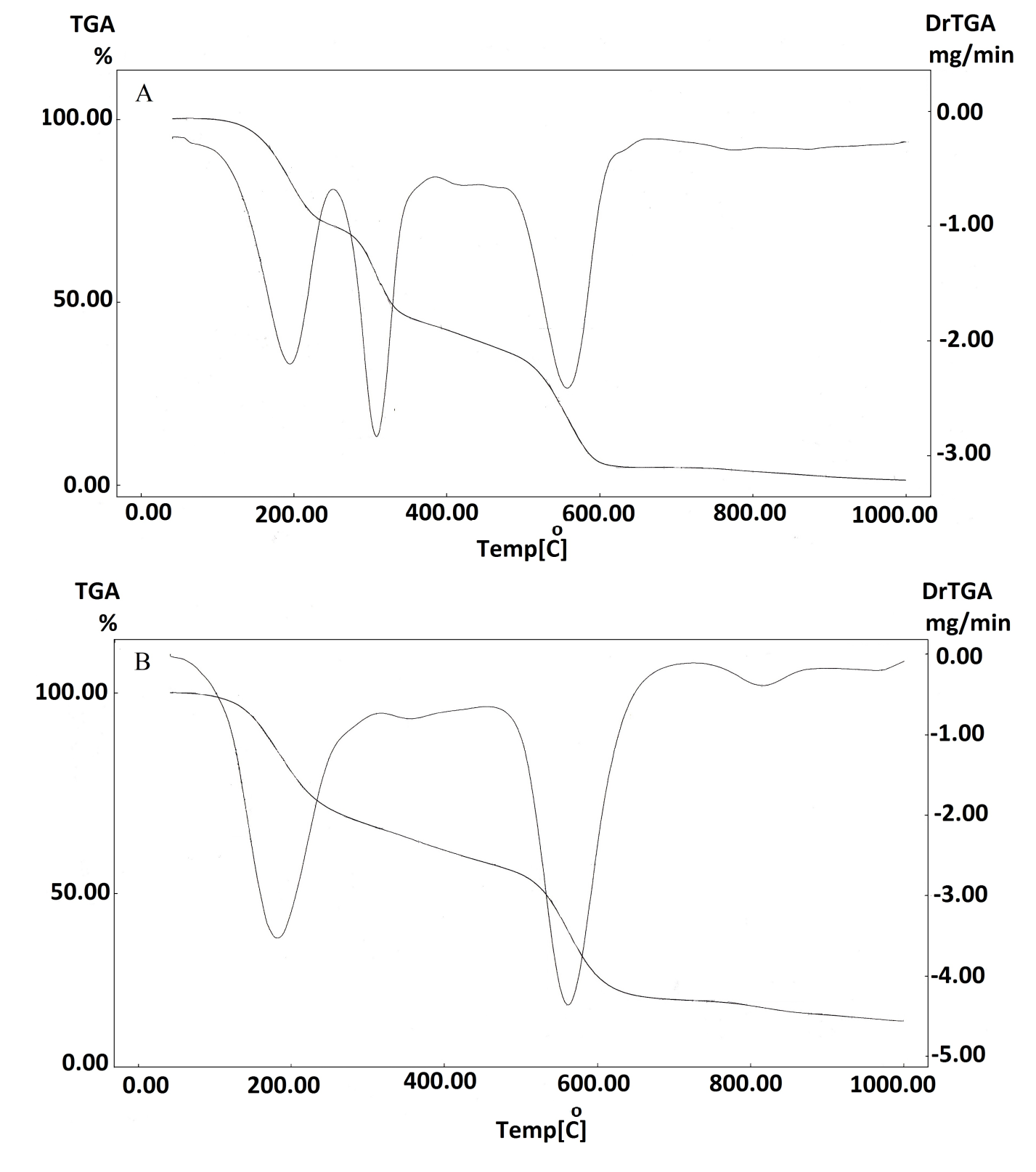


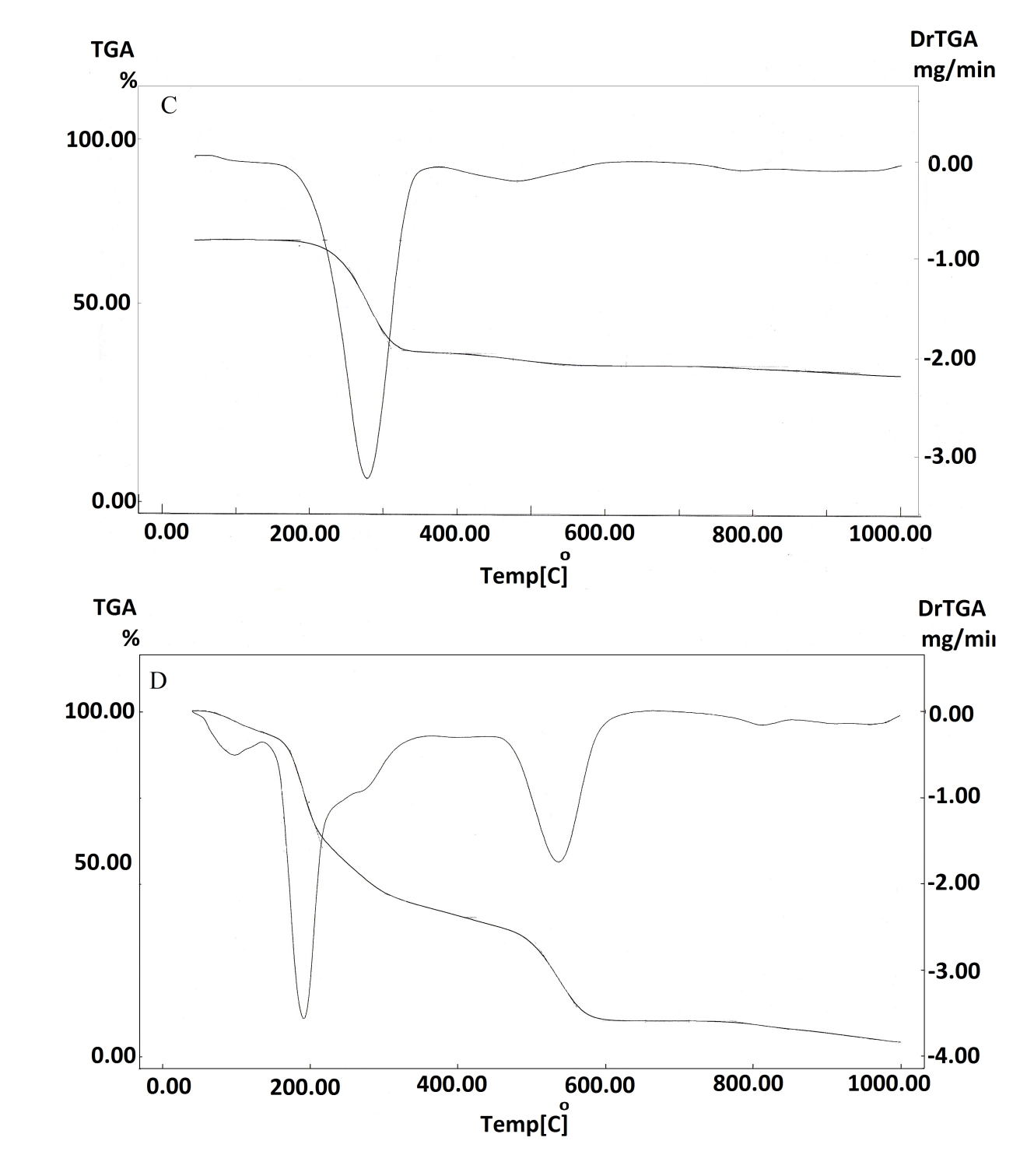


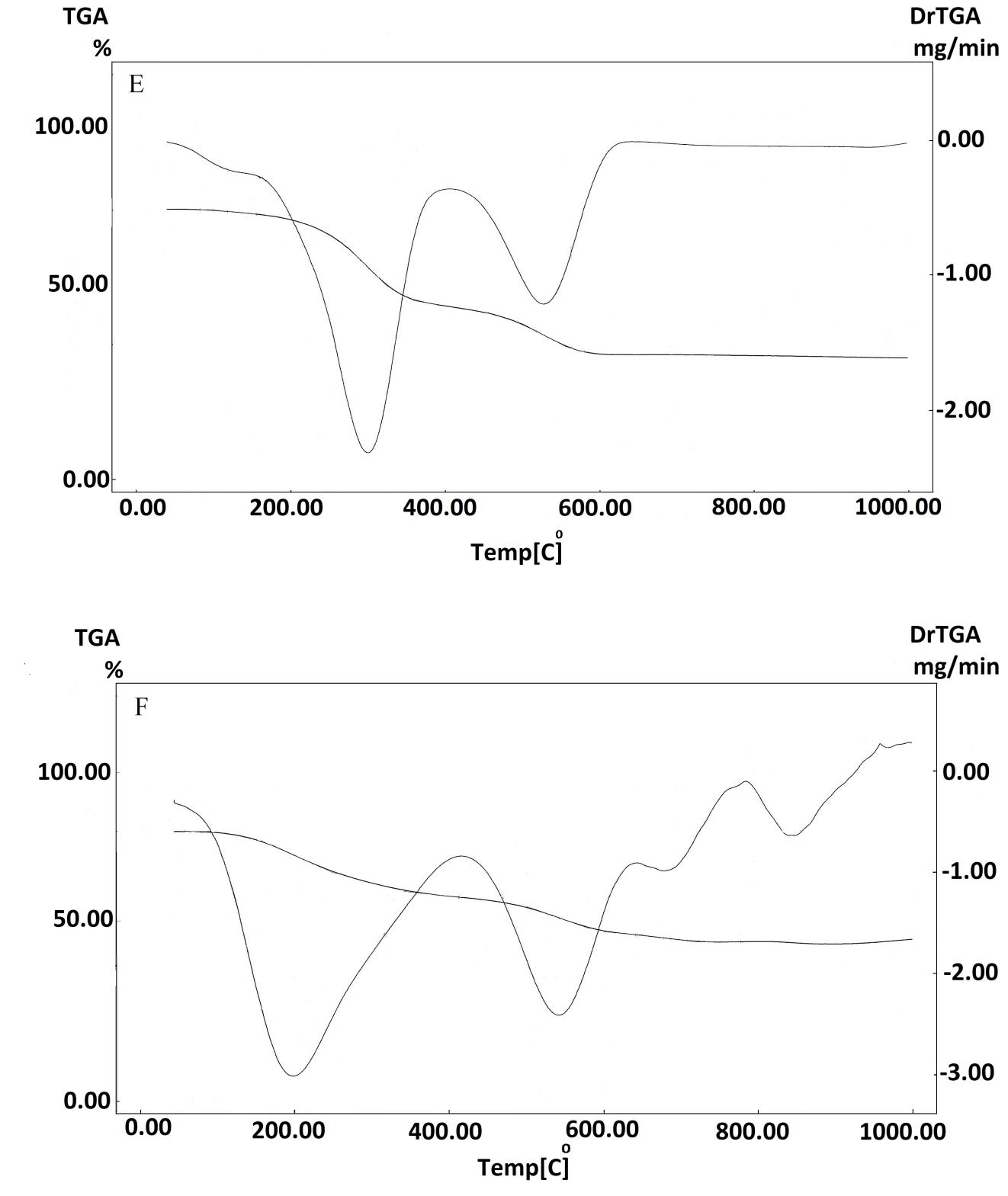


**Fig S1:** TGA and DTG diagrams for (A) L_1_, (B) [Ag(L_1_)_2_(H_2_O)_2_]NO_3_, (C), L_2_ (D) [Ag(L_2_)_2_(H_2_O)_2_]NO_3_.H_2_O, (E) L_3_ and (F) [Ag(L_3_)_2_(H_2_O)_2_]NO_3_.


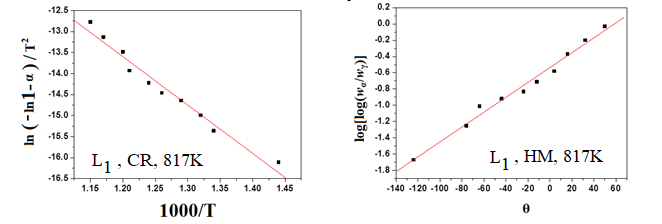


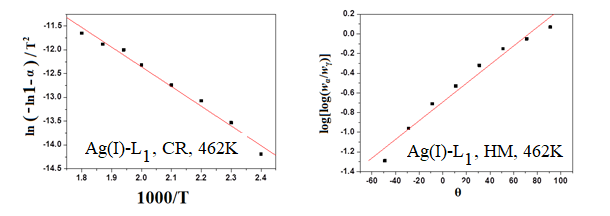


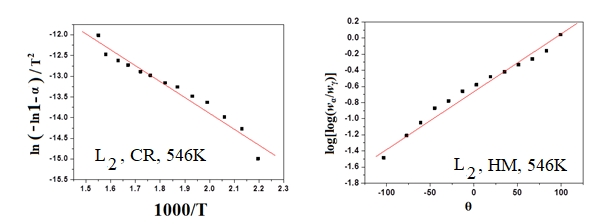


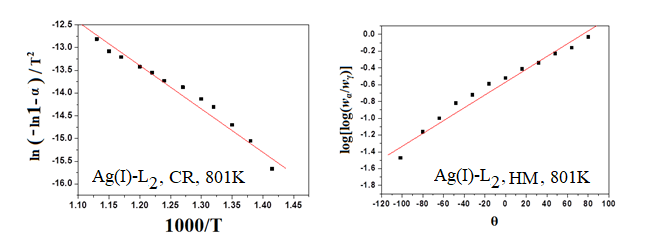


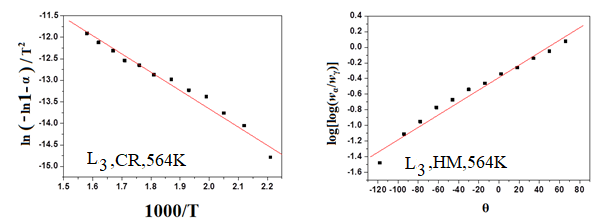


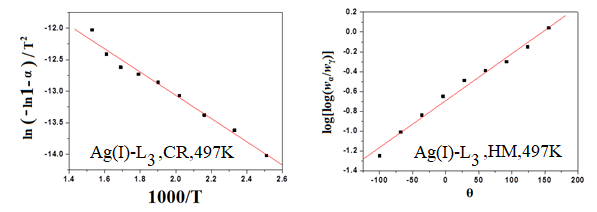


**Fig. S2:** The diagrams of kinetic parameters of L_1_, [Ag(L_1_)_2_(H_2_O)_2_]NO_3_, L_2_, [Ag(L_2_)_2_(H_2_O)_2_]NO_3_.H_2_O, L_3_ and [Ag(L_3_)_2_(H_2_O)_2_]NO_3_using Coats-Redfern (CR) and Horowitz-Metzger (HM) equations.

**Scheme S1**: Fragmentation pattern of [Ag(L_1_)_2_(H_2_O)_2_]NO_3_

**Scheme S2**: Fragmentation pattern of [Ag(L_2_)_2_(H_2_O)_2_]NO_3_.H_2_O

**Scheme S3**: Fragmentation pattern of [Ag(L_3_)_2_(H_2_O)_2_]NO_3_
